# Evaluation of Mammalian and Intermediate Host Surveillance Methods for Detecting Schistosomiasis Reemergence in Southwest China

**DOI:** 10.1371/journal.pntd.0000987

**Published:** 2011-03-08

**Authors:** Elizabeth J. Carlton, Michael N. Bates, Bo Zhong, Edmund Y. W. Seto, Robert C. Spear

**Affiliations:** 1 Division of Environmental Health Sciences, School of Public Health, University of California, Berkeley, California, United States of America; 2 Division of Epidemiology, School of Public Health, University of California, Berkeley, California, United States of America; 3 Institute of Parasitic Diseases, Sichuan Center for Disease Control and Prevention, Chengdu, China; Imperial College London, United Kingdom

## Abstract

**Background:**

Schistosomiasis has reemerged in China, threatening schistosomiasis elimination efforts. Surveillance methods that can identify locations where schistosomiasis has reemerged are needed to prevent the further spread of infections.

**Methods and Principal Findings:**

We tested humans, cows, water buffalo and the intermediate host snail, *Oncomelania hupensis*, for *Schistosoma japonicum* infection, assessed snail densities and extracted regional surveillance records in areas where schistosomiasis reemerged in Sichuan province. We then evaluated the ability of surveillance methods to identify villages where human infections were present. Human infections were detected in 35 of the 53 villages surveyed (infection prevalence: 0 to 43%), including 17 of 28 villages with no prior evidence of reemergence. Bovine infections were detected in 23 villages (infection prevalence: 0 to 65%) and snail infections in one village. Two common surveillance methods, acute schistosomiasis case reports and surveys for *S. japonicum*-infected snails, grossly underestimated the number of villages where human infections were present (sensitivity 1% and 3%, respectively). Screening bovines for *S. japonicum* and surveys for the presence of *O. hupensis* had modest sensitivity (59% and 69% respectively) and specificity (67% and 44%, respectively). Older adults and bovine owners were at elevated risk of infection. Testing only these high-risk human populations yielded sensitivities of 77% and 71%, respectively.

**Conclusions:**

Human and bovine schistosomiasis were widespread in regions where schistosomiasis had reemerged but acute schistosomiasis and *S. japonicum*-infected snails were rare and, therefore, poor surveillance targets. Until more efficient, sensitive surveillance strategies are developed, direct, targeted parasitological testing of high-risk human populations should be considered to monitor for schistosomiasis reemergence.

## Author Summary

Schistosomiasis has reemerged in China in regions where it was previously controlled. As reductions in schistosomiasis, a water-born parasitic infection, prompt consideration of schistosomiasis elimination, surveillance strategies that can signal reemergence and prevent further lapses in control are needed. We examined the distribution of *Schistosoma japonicum*, the species that causes schistosomiasis in China, in 53 villages. The villages were located in regions of Sichuan province where schistosomiasis reemergence had been documented by public health authorities. We tested three key reservoirs, humans, cows and water buffalo, and freshwater snails for *S. japonicum* infection in an effort to identify high-risk populations and evaluate their ability to signal reemergence. Human and bovine infections were common, detected in 35 villages and 23 villages, respectively, but infected snails were rare, found in only one village. Two commonly used surveillance methods, hospital reports of acute schistosomiasis and surveys for *S. japonicum*-infected snails, grossly underestimated the number of villages where human infections were present. Schistosomiasis was widespread in the region we studied, highlighting the danger reemergence poses to disease elimination programs. Surveillance systems that monitor high-risk populations such as older adults or bovine owners should be considered to promote detection of reemergence.

## Introduction

The success of disease control programs in reducing schistosomiasis infections and morbidity have prompted consideration of the elimination of human schistosomiasis [1], [2]. Dramatic declines in *Schistosoma haematobium* and *S. mansoni* have been observed following widespread distribution of the antihelminthic drug, praziquantel, in six countries in sub-Saharan Africa [3]–[5]. Disease control efforts in China, including a ten-year partnership with the World Bank to promote treatment, have led to the interruption of *S. japonicum* transmission in 5 of 12 endemic provinces and 60% of endemic counties [6], [7]. Currently, China is aiming to eliminate schistosomiasis, setting an initial goal of reducing human and bovine infection prevalence below 1% in every endemic region by 2015 [8]. If successful, China's program may serve as a model for schistosomiasis control elsewhere.

However, schistosomiasis has reemerged in previously controlled regions, highlighting the challenges of sustaining reductions in infections. In Sichuan, China, schistosomiasis was identified in 8 of 46 counties that had met Chinese Ministry of Health criteria for transmission control, which require the reduction of human and bovine infection prevalence below 1% in every endemic village [9]. Nationwide, 38 counties that have met transmission control criteria have been reclassified as reemerging [7]. In the absence of a vaccine or lasting immunity, and with at least forty competent mammalian reservoirs, *S. japonicum* reemergence remains a threat in controlled regions [10]. Little is known about the epidemiology of reemerging schistosomiasis, including how infections are distributed across human populations, other mammalian reservoirs and intermediate host snails.

Surveillance systems that can identify areas where lapses in control have occurred are an essential component of disease elimination strategies [11], [12]. They can enable timely treatment of infected populations and interventions to prevent further spread of infections. In China, surveillance for schistosomiasis in controlled regions includes hospital-based surveillance for acute schistosomiasis, surveys for the intermediate host snail, *Oncomelania hupensis*, and direct testing of the human population [13]. Acute schistosomiasis is triggered by the migration of the parasite through the body shortly after infection, leading to rapid onset of symptoms including high fever, myalgia and eosinophilia [14]. Due to the quick and severe onset, acute schistosomiasis, which is a reportable disease in China, can serve as a sentinel event, signaling the reemergence or emergence of schistosomiasis, as occurred in the Yangtze River valley following flooding events and in Sichuan province [9], [15], [16]. But acute schistosomiasis is rare, comprising less than 1% of all schistosomiasis cases [17]. It is possible for schistosomiasis to reemerge more quietly, as schistosomiasis typically induces chronic morbidity [18]–[20], leading to uncertainties about the sensitivity of surveillance methods that rely on acute schistosomiasis case reports. Similarly, surveillance for schistosome-infected snails and children is recommended in regions approaching schistosomiasis elimination, but how well these surveillance targets can identify areas where human infections are present remains uncertain [21].

In an effort to inform surveillance for *S. japonicum* reemergence, we examined the distribution of *S. japonicum* infections in human, bovine and snail populations in regions where schistosomiasis had reemerged. We then evaluated the ability of active and passive surveillance methods, including acute schistosomiasis case reporting, surveys for the presence of *O. hupensis*, and surveys for *S. japonicum* infections in *O. hupensis*, cows, water buffalo, and high-risk human populations, to identify villages where human infections were present.

## Methods

Three of the eight counties where schistosomiasis reemergence was detected in Sichuan province following transmission control were selected for inclusion in this study [9]. Counties were selected based on the availability of surveillance records and the willingness of the control station personnel to collaborate on this project. Due to the sensitive nature of conducting infection surveys in regions where schistosomiasis transmission control criteria have officially been met and to promote candid reporting, the names and exact locations of the counties and study villages have been withheld.

County surveillance records were examined in March 2007 in order to identify all villages where *S. japonicum* had been detected after the attainment of transmission control. We examined reemergence at the smallest community unit, the natural village or production group (referred to here as, simply, *village*) which generally includes 100 to 200 residents. Reemergence criteria were based on typical post-control surveillance. This includes passive monitoring for acute schistosomiasis through health providers, and active surveillance for *S. japonicum*-infected snails and humans through surveys conducted at least once every three years in formerly endemic areas. Human surveys in controlled areas often focus on children, as *S. japonicum* infections in children who were born after transmission control was attained indicate renewed transmission. Records were examined from the year that transmission control was attained through March 2007.

We classified villages as historically reemerging (HR) if schistosomiasis was endemic prior to transmission control and acute human schistosomiasis, a *S. japonicum-*infected snail or a *S. japonicum* infection in a child younger than 12 years was detected after transmission control. County surveillance records were supplemented by provincial surveillance data when available. We identified 112 HR villages, including 109 villages identified through county surveillance records and three villages identified through provincial records (Table 1). Infected snails were the most frequent indicator of reemergence. Acute schistosomiasis and infected children were less commonly detected. Evidence of reemergence was first detected in County 1 five years after transmission control criteria were met, the same year as reemergence was detected in County 2, which borders County 1 and had attained transmission control 15 years earlier.

**Table 1:** Indicators of reemergence in 112 villages in three counties in Sichuan, China.

	Total	County 1	County 2	County 3
Villages where surveillance records indicated…				
Acute schistosomiasis	25	16	1	8
Infected snails	100	47	23	30
Infected children	7	4	0	3
Any of the above	112	47	24	41
Year attained transmission control		1995	1985	1987
Year reemergence first detected		2000	2000	1997

County and provincial surveillance were examined from the year transmission control was attained through March 2007.

Twenty-five villages were selected from the HR villages. Villages were stratified by county and how reemergence was indicated and a sample was selected from each stratum. The selected villages include 11 villages with acute schistosomiasis, 19 with infected snails and one village with infected children (five villages had multiple indicators), detected from 1997 through 2005. For comparison, 28 villages were selected from formerly endemic villages in the same counties with no history of reemergence (NHR) since transmission control was achieved. In each of the 53 selected villages, village residents were interviewed to describe demographic and household characteristics, and human, bovine and snail infection surveys were conducted.

### Census and interviews

In June 2007, all residents aged six years and older in the 53 selected villages were invited to complete a brief survey about their age, sex, occupation, highest level of schooling, travel and schistosomiasis treatment history. The head of each household was also asked to complete a detailed questionnaire describing household agricultural practices, ownership of domestic animals and socioeconomic indicators. Given the challenges of estimating income in agrarian regions, household socioeconomic status was assessed based on household assets [22]. The head of each household was asked if any member of his or her household owned a car, tractor, motorcycle, computer, television, washing machine, air conditioner or refrigerator. A household asset score was assigned based on the number of assets owned. In 2008, attempts were made to interview any participant in the human infection survey missing household or individual interview data from 2007.

Questionnaires were pilot-tested to ensure questions were appropriate for the study region. Interviews were conducted by trained staff at the Institute of Parasitic Diseases (IPD), Sichuan Center for Disease Control and Prevention and the county Anti-schistosomiasis Control Stations fluent in Sichuan dialect, which is spoken by the study population. Household and individual interviews were scanned using optical mark recognition software (Remark Office OMR, Gravic Corporation, Malvern, PA). Approximately 10% of scanned questionnaires were checked against paper records to ensure data accuracy.

### Human infection surveys

In November and December 2007, all residents aged 6 to 65 years were invited to submit three stool samples from three consecutive days which were analyzed using the miracidium hatching test and the Kato-Katz thick smear procedure [23], [24]. Of 3,009 participants, three stool samples were collected from 2,504 participants (85%), two samples from 7% and one sample from 8%. Samples were collected from villages daily and brought to a central laboratory in each county where they were stored out of direct sunlight until processing (90% were processed within one day of collection).

The miracidium hatching test was used to examine each stool sample. Approximately 30 g of stool were suspended in aqueous solution, strained with copper mesh to remove large particles, then strained with nylon mesh to concentrate schistosome eggs. This sediment was re-suspended and left in a room with ambient temperatures between 28 and 30°C. Two, five and eight hours after preparation, samples were examined for the presence of miracidia for at least two minutes each time.

Using the Kato-Katz thick smear procedure, three slides were prepared using 41.7 mg of homogenized stool from the first sample submitted by each participant. Three slides were prepared for 97% of infection survey participants (two slides were prepared for 21 participants, one slide was prepared for 21 participants and no slides were prepared for 55 participants). Each slide was examined using a dissecting microscope and if any *S. japonicum* eggs were detected, the species and number of eggs was confirmed by a second reader. Infection intensity, expressed in eggs per gram of stool (EPG), was calculated as the total number of *S. japonicum* eggs divided by the total sample weight. A person was classified as infected if the miracidium hatching test was positive or at least one egg was detected using the Kato-Katz technique.

### Bovine infection surveys

The domestic bovines, water buffalo (*Bubalus bubalis*) and cows (*Bos taurus*), were tested for *S. japonicum* infection at the same time as the human surveys. Attempts were made to collect three stool samples from all bovines in study villages by keeping the animal in a pen or tied until stool was produced on three separate days. Samples were collected shortly after defecation and from the center of fresh stool samples in order to minimize potential contamination. Three samples were collected from 68% of the 537 bovines tested, two samples from 18% and one sample from 14%. Each sample was examined using the miracidium hatching test as described above. Due to the rapid hatching and short survival of miracidia in bovine stool, samples were examined one, three and five hours after preparation and 99% of samples were processed within one day of collection. Bovines were classified as infected if at least one hatching test was positive for *S. japonicum*.

Bovines with at least one positive hatching test were subsequently examined using an adaptation of the Danish Bilharziasis Laboratory (DBL) method in order to estimate infection intensity [25]. Briefly, 5 g of homogenized stool were washed through a series of three sieves (mesh size: 400 µm, 100 µm and 45 µm). The material in the 45 µm sieve was washed into a sedimentation tube, two drops of formalin were added and the suspension was left in the dark to sediment. The solution was centrifuged and the top half of the liquid gently decanted. The remaining sediment was re-suspended, adding enough water to create 10 mL of solution, re-centrifuged, and the top 80% of the solution gently decanted in order to obtain 2 mL of solution. A thick smear approach was used to count the eggs. Infection intensity, expressed in EPG, was calculated as the total number of eggs divided by the sample weight.

### Snail infection surveys

In April 2007, all irrigation ditches in the study villages were surveyed for *O. hupensis*. Teams of trained IPD and county Anti-Schistosomiasis Control Station staff with extensive experience conducting snail surveys collected samples at 10 m intervals along irrigation ditches. At each location, a square frame (*kuang*) measuring 0.11 m^2^ was placed at the waterline and all *O. hupensis* snails within the frame were collected. In addition, snails were sampled from 10 terrace walls per village (or all terrace walls if there were fewer than 10 terraces), since the lower portions of terrace walls accumulate moisture and may provide suitable habitat for snails. The sampling frame was placed at the base of the terrace walls in three locations: the middle and both ends of the terrace and all snails within the frame were collected. Collected snails were deposited in paper envelopes and brought to the laboratory where they were crushed between two glass slides and inspected for cercariae using a dissecting microscope.

### Ethical approval and treatment

All adult participants provided written, informed consent before participating in this study. All children provided assent and their parents or guardians provided written, informed permission for them to participate in this study. The research protocol was approved by the Sichuan Institutional Review Board and the University of California, Berkeley, Committee for the Protection of Human Subjects. Each person who tested positive for *S. japonicum* was provided treatment with 40 mg per kg of praziquantel tablets by the county Anti-Schistosomiasis Control Station. Because bovine stool samples were collected after they were excreted, the Animal Care and Use Committee at the University of California, Berkeley determined the protocol was exempt from review. All bovines testing positive were referred to the county veterinary station for treatment with praziquantel. The reporting of this cross-sectional study was evaluated using the Strengthening the Reporting of Observational Studies in Epidemiology (STROBE) checklist (Checklist S1).

### Statistical analysis

In order to identify subpopulations at high risk of *S. japonicum* infection in reemerging areas, human infection prevalence and intensity were examined across 11 demographic variables: age, sex, occupation, educational attainment, socioeconomic status, time spent out of the village, bovine ownership and whether members of the household plant rice, corn, wheat or rapeseed. Similarly, bovine infection prevalence was examined across species, sex and age (as reported by owner). Variables were selected based on their potential to affect exposure to *S. japonicum*, and the ease with which surveillance teams could identify individuals based on the selected characteristics. For each demographic variable, we estimated an odds ratio (OR) and 95% confidence interval (CI) using logistic regression, modeling infection status as a function of the demographic variable, adjusting for county of residence and village HR status. For humans, we also tested the hypothesis that, among the infected, infection intensity is predicted by these same 11 demographic variables. We estimated the arithmetic mean EPG for each subpopulation. We modeled infection intensity, in EPG, as a negative binomial distribution because even among the infected, infection intensity was overdispersed as is common for helminthic infections (note that 55% of those who tested positive for *S. japonicum* had zero detectable eggs, testing positive by the miracidium hatching test only). Logistic and negative binomial models accounted for correlation of infections within villages using generalized estimating equations (GEE) with exchangeable correlation and with inference from robust variance estimates [26], [27].

We evaluated five types of schistosomiasis surveillance methods for their ability to identify villages where human infections were present: acute schistosomiasis case reports, surveys for the presence of *O. hupensis*, and surveys for *S. japonicum* infections in *O. hupensis*, bovines and high-risk human populations. The sensitivity and specificity of each method was calculated using the human infection survey results as the gold standard: villages where at least one person tested positive for *S. japonicum* were classified as true positives, villages where all people tested negative for *S. japonicum* were classified as true negatives. Bootstrapping was used to estimate the variability around each point estimate [28]. We drew 1,000 random samples of the 53 villages with replacement, estimated the sensitivity and specificity for each sampled population, and the 2.5^th^ and 97.5^th^ percentile values were used to generate nonparametric 95% CIs. The sensitivity and specificity of testing high-risk human populations were estimated for characteristics that were significant predictors of human infection status and that defined less than 50% of the population.

The village selection procedure involved an oversampling of villages where surveillance records indicated reemergence generally and where acute cases had been reported, in particular. Failure to account for this selection procedure can lead to biased estimates of the sensitivity and specificity of acute case reporting. We therefore applied differential weights in estimating the sensitivity and specificity of acute case reporting. Villages where acute cases were present were weighted by 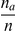 where *n* is the total number of villages formerly endemic for schistosomiasis and *n_a_* is equal to 25, the number of villages where one or more acute schistosomiasis cases were reported in the three counties. Villages where acute cases were not present were divided into two subgroups: HR villages where no acute cases were found, which were weighted using 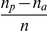, and NHR villages, weighted using 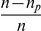 where *n_p_* is equal to 112, the number of formerly endemic villages where surveillance records indicated reemergence. Both *n_p_* and *n_a_* were directly measured through our examination of surveillance records. The number of previously endemic villages, *n*, is not known precisely and was estimated, based on conversations with county schistosomiasis control officers, to be 1,000. Given the uncertain value of *n*, we conducted a sensitivity analysis, estimating sensitivity and specificity setting *n* to lower and upper bound estimates (500 and 5,000 villages, respectively).

Tests of statistical significance were conducted setting α  =  0.05. Data analysis was conducted using Stata, version 10 (StataCorp, College Station, TX, USA) and R, version 2.9.2 (www.r-project.org).

## Results

### Characteristics of the 53 surveyed villages

Beginning in June 2007, we enrolled 4,399 study participants in 53 villages and 1,784 heads of household completed the household questionnaire. There were, on average, 83 participants per village (range: 30, 169).

Most adults (≥18 years) were farmers (96.5%) as shown in Table 2. Rice and corn were the principal crops planted in the summer months (typically, May to September), whereas rapeseed and wheat were the major crops planted during the winter growing season (typically, October to April). Socioeconomic status was modest: 61.3% of individuals reported their household owned no more than two of eight specified household assets. The most commonly reported asset was a television, owned by 94.4% of households, followed by a washing machine (55.5%), motorcycle (33.1%) and refrigerator (16.5%). Tractors (2.8%), air conditioners (2.0%), cars (1.7%) and computers (0.5%) were rare.

**Table 2:** Characteristics of residents in 53 villages in reemerging regions of Sichuan, China.

	Total population identified in census		Tested for *S. japonicum* infection	
	No.	(%)	No.	(%)
County				
County 1	1,798	(40.9)	1,233	(41.0)
County 2	1,200	(27.3)	877	(29.1)
County 3	1,401	(31.8)	899	(29.9)
Historically reemerging village				
No	2,335	(52.0)	1,566	(53.1)
Yes	2,064	(48.0)	1,443	(46.9)
Sex				
Female	2,158	(50.2)	1,500	(51.6)
Male	2,138	(49.8)	1,406	(48.4)
Age				
<12 years	332	(7.8)	222	(7.7)
12–17 years	357	(8.3)	121	(4.2)
18–29 years	445	(10.4)	181	(6.3)
30–39 years	899	(21.0)	578	(20.0)
40–49 years	878	(20.5)	659	(22.8)
≥50 years	1,368	(32.0)	1,130	(39.1)
Occupation*				
Farmer	3,457	(96.5)	2,502	(98.3)
Other†	126	(3.5)	43	(1.7)
Household grows				
Corn	1,683	(39.8)	1,208	(42.6)
Rapeseed	3,625	(85.8)	2,452	(86.4)
Rice	3,526	(83.5)	2,357	(83.0)
Wheat	2,330	(55.1)	1,618	(57.0)
Household owns bovines				
No	2,738	(64.8)	1,783	(62.8)
Yes	1,486	(35.2)	1,055	(37.2)
Household asset score				
0–1 assets	1,280	(30.3)	869	(30.6)
2 assets	1,309	(31.0)	904	(31.8)
3 assets	1,078	(25.5)	697	(24.6)
4+ assets	558	(13.2)	369	(13.0)
Education*				
Elementary school or less	2,314	(64.7)	1,752	(69.1)
Middle school	1,063	(29.7)	674	(26.6)
High school or more	197	(5.5)	110	(4.3)
Days out of the village last year				
Did not leave	2,576	(65.5)	1,869	(72.2)
1–30 days	314	(8.0)	144	(5.6)
≥31 days	1,040	(26.5)	577	(22.3)
Took praziquantel in the past year				
No	2,344	(57.7)	1,476	(55.3)
Yes	1,717	(42.3)	1,195	(44.7)

*Includes only adults (≥18 years).

**†:** Other occupations include laborer (67), student (36), business person (14), government official (6) and fisherman (3).

**Figure 1:**
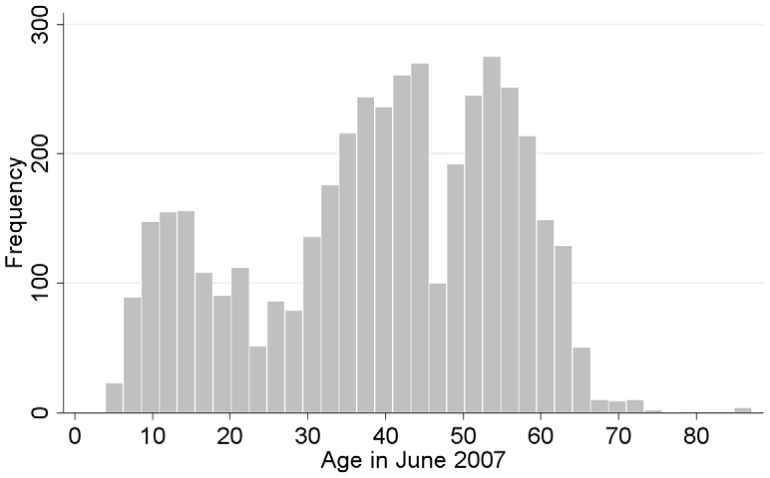
The age distribution of 4,279 residents from 53 villages in regions where schistosomiasis reemerged.

There were few teenagers and young adults among the study population relative to other age groups (Figure 1). Residents reported many people, particularly younger populations, had left their villages to find work in urban areas. There is also a dip in the population corresponding with the birth years 1959 to 1961, the years of the Chinese famine. Travel outside of the village was common among study participants. Most young adults (aged 18–29 years) reported spending at least one month out of their village in the past year, as did 40% of adults aged 30–39 years and 26% of adults aged 40–49 years. Most of these individuals left to work as laborers. Some teenagers (aged 12–17 years) also reported living outside of their village for more than one month in the past year (21%), primarily to attend school.

Schistosomiasis control was ongoing in the study regions and occurred in both HR and NHR villages. Praziquantel treatment in 2006 or 2007 (prior to the infection survey) was reported by 45% of residents in HR villages and 40% of residents in NHR villages. County records indicated that the molluscicide, niclosamide, was applied to snail habitats in 23 of the 25 HR villages and 25 of the 28 NHR villages in 2006.

### Human *S. japonicum* infection

We tested 3,009 people from the 53 villages for *S. japonicum* infection. Participation in the infection surveys ranged from 45% to 97% by village and varied by county, age, occupation and travel out of the village (Table 2). Participation was lowest for individuals aged 12–29 years.

**Figure 2:**
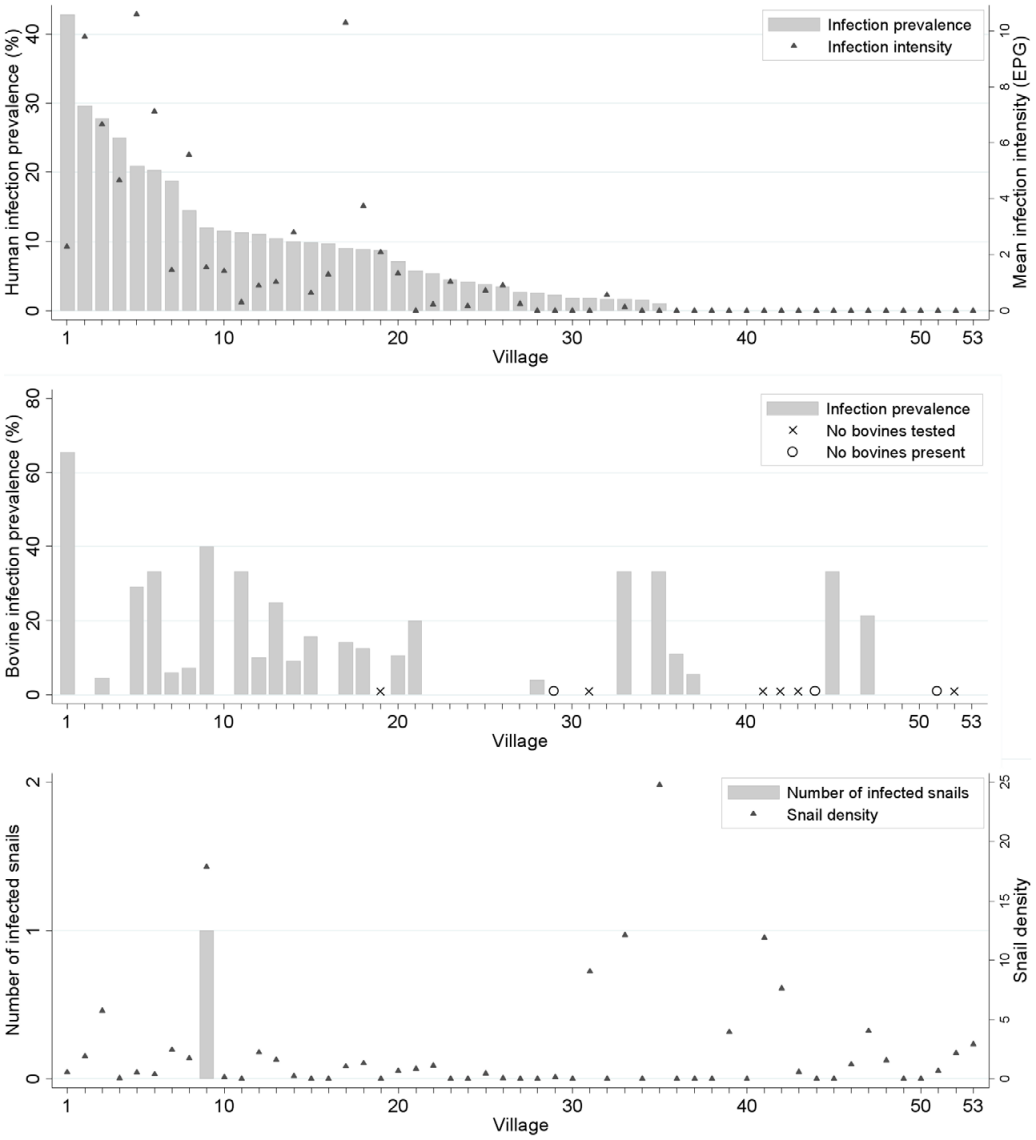
The distribution of *S. japonicum* infections in humans (top), bovines (middle) and snails (bottom). The 53 villages surveyed are located in regions of Sichuan, China where schistosomiasis has reemerged. Human and bovine *S. japonicum* infections were measured in November and December 2007. Snail density and *S. japonicum* infection status were measured in April 2007. Snail density is the number of *O. hupensis* per m^2^ of irrigation ditch.

In 2007, 195 people (6.5%) tested positive for *S. japonicum*, including 159 who tested positive using the miracidium hatching test and 88 who tested positive using the Kato-Katz thick smear procedure. Human infections were detected in 35 villages, including 18 of the 25 HR villages, and in 17 of the 28 NHR villages. Human infection prevalence ranged from 0 to 42.9% by village (Figure 2), and varied by county and HR status (Table 3). Mean infection intensity was 1.6 EPG, and the maximum village infection intensity was 10.6 EPG. *S. japonicum* eggs were clustered in a few individuals: 24% of all eggs detected were excreted by two individuals.

**Table 3:** Predictors of human *S. japonicum* infection in regions of Sichuan, China where schistosomiasis reemerged.

	*S. japonicum* infection prevalence			*S. japonicum* infection intensity		
	Tested	% inf.	OR (95% CI)*	Infected	Mean EPG	RR (95% CI)*
**Regional variables**						
County						
County 1	1,233	8.76	1.00	106	27.74	1.00
County 2	877	7.30	0.78 (0.33–1.85)	64	18.25	0.66 (0.30–1.49)
County 3	899	2.56	0.20 (0.08–0.53)	23	20.87	0.75 (0.31–1.82)
Historically reemerging village						
No	1,566	4.09	1.00	64	27.69	1.00
Yes	1,443	9.08	2.93 (1.45–5.92)	129	21.83	0.73 (0.29–1.85)
**Demographic variables**						
Sex						
Female	1,500	6.13	1.00	92	25.83	1.00
Male	1,406	7.04	1.18 (0.91–1.55)	97	22.19	0.89 (0.35–2.26)
Age†						
<12 years	222	2.70	1.00	6	0.00	
12–17 years	121	4.96	1.93 (0.54–6.93)	6	13.33	
18–29 years	181	4.42	1.42 (0.52–3.89)	8	20.00	
30–39 years	578	7.09	2.48 (0.90–6.83)	41	22.63	
40–49 years	659	6.98	2.48 (0.92–6.70)	46	39.30	
50+ years	1,130	7.43	2.73 (1.04–7.20)	82	18.93	
Occupation†,‡						
Farmer	2,502	7.03	1.00	174	25.56	
Other	43	6.98	0.76 (0.24–2.39)	3	0.00	
Household grows corn						
No	1,631	4.97	1.00	80	21.90	1.00
Yes	1,208	8.94	1.31 (0.92–1.86)	107	23.93	1.96 (0.85–4.51)
Household grows rapeseed						
No	387	2.84	1.00	10	20.00	1.00
Yes	2,452	7.26	1.71 (1.02–2.89)	177	23.23	1.18 (0.63–2.21)
Household grows rice						
No	482	4.98	1.00	24	25.67	1.00
Yes	2,357	7.00	0.91 (0.40–2.09)	163	22.67	0.43 (0.09–2.16)
Household grows wheat						
No	1,221	6.47	1.00	78	32.10	1.00
Yes	1,618	6.80	1.15 (0.82–1.61)	109	16.59	0.54 (0.17–1.68)
Household owns bovines						
No	1,783	4.21	1.00	74	26.70	1.00
Yes	1,055	10.81	1.38 (1.01–1.90)	113	20.67	0.82 (0.35–1.95)
Household asset score						
0–1 assets	869	8.17	1.00	70	17.94	1.00
2 assets	904	7.08	1.05 (0.66–1.68)	63	31.49	1.29 (0.45–3.67)
3 assets	697	5.16	0.81 (0.47–1.41)	36	21.33	0.94 (0.46–1.94)
4+ assets	369	4.88	0.91 (0.52–1.58)	18	16.89	0.63 (0.16–2.45)
Education‡						
Elementary school or less	1,752	7.93	1.00	137	25.87	1.00
Middle school	674	5.19	0.64 (0.46–0.89)	35	21.49	0.78 (0.35–1.75)
High school or more	110	3.64	0.37 (0.15–0.90)	4	20.00	0.72 (0.35–1.47)
Days out of the village last year						
Did not leave	1,869	6.37	1.00	119	17.01	1.00
1–30 days	144	6.94	1.39 (0.78–2.48)	10	14.40	0.74 (0.27–2.05)
31+ days	577	6.07	0.82 (0.62–1.09)	35	34.74	1.48 (0.43–5.04)

Human infections were measured in November and December 2007 in 53 villages.

*Odds ratios and relative risks for each demographic variable were calculated adjusting for county and historical evidence of reemergence in the village. Village-level correlation was accounted for using generalized estimating equations.

**†:** Models of *S. japonicum* infection intensity could not be fitted because no eggs were detected in some groups.

**‡:** Includes only adults (≥18 years).

We identified several groups at high risk of infection on the basis of demographic characteristics (Table 3). Infection prevalence was highest in adults, aged 50 years and older, individuals living in households planting rapeseed, individuals in households with one or more bovines and adults with no formal schooling beyond elementary school. These variables were significant predictors of infection status, controlling for county and village HR status. Because most residents in our study region lived in households planting rapeseed (86%) and were adults with less than an elementary school education (58%), only bovine ownership and older age were examined as target populations for human surveillance (comprising 37% and 39% of the population, respectively). Human *S. japonicum* infections did not vary by sex, occupation, household asset score or time spent out of the village.

Among the infected, infection intensity did not vary significantly by any of the demographic characteristics examined. Infection intensity did increase with age but was highest in adults aged 40 to 49 years, rather than the oldest, most frequently infected age group.

### Bovine *S. japonicum* infection

We identified 821 cows and water buffalo present in 50 of the 53 villages. We tested 537 (65.4%) bovines from 44 villages for *S. japonicum* infection. The six villages where bovines were present but infection surveys were not conducted had fewer bovines (mean 5.8 bovines, range: 3, 8) compared to villages where infection surveys were conducted (mean 17.9 bovines, range: 2, 42).

Bovine infections were detected in 23 villages, including 15 of the 19 HR villages where bovines were tested and 8 of the 25 NHR villages where bovines were tested. Mean bovine infection prevalence was 13.4%, with a maximum village infection prevalence of 65.4% (Figure 2). Like human infection prevalence, bovine infection prevalence varied by county and village HR status (Table 4). We measured infection intensity in 67 of the 72 bovines positive by the miracidium hatching test: 11 had detectable eggs. While bovine infection prevalence was higher than human infection prevalence, mean bovine infection intensity was lower, ranging from 0 to 0.11 EPG by village.

**Table 4:** Predictors of bovine *S. japonicum* infection in regions of Sichuan, China where schistosomiasis reemerged.

	*S. japonicum* infection prevalence		
	Tested	% inf.	OR (95% CI)*
**Regional variables**			
County			
County 1	213	8.0	1.00
County 2	290	16.6	2.36 (0.87–6.36)
County 3	34	20.6	2.67 (0.89–8.04)
Historically reemerging village			
No	256	5.1	1.00
Yes	281	21.0	4.15 (1.74–9.87)
**Demographic variables**			
Bovine type			
Water buffalo	117	10.3	1.00
Cow	407	14.3	1.14 (0.46–2.81)
Sex			
Female	360	15.8	1.00
Male	54	18.5	1.17 (0.59–2.31)

Bovine infections were measured in November and December 2007 in 44 villages.

*Odds Ratios for each demographic variable were calculated adjusting for county and historical evidence of reemergence in the village. Village-level correlation was accounted for using generalized estimating equations.

**Figure 3:**
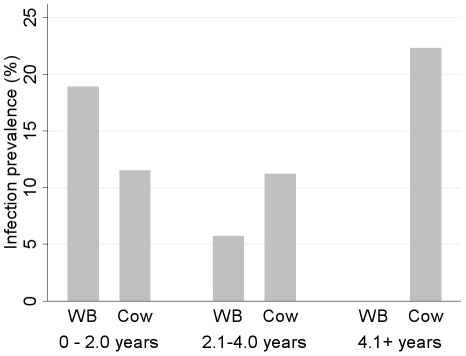
The prevalence of *S. japonicum* infections by age in bovines in regions where schistosomiasis reemerged. Bovine infections were measured in November and December 2007 in 44 villages in Sichuan, China. None of the 18 water buffalo aged 4 years and older were infected.

Infection prevalence was modestly but not significantly higher in cows compared to water buffalo, adjusting for county and village HR status (Table 4). Bovine sex did not predict infection status. Water buffalo infections declined with age whereas cow infections increased with age (Figure 3).

### Snail *S. japonicum* infection

We surveyed a total of 15,054 locations along irrigation ditches and 2,498 locations at the base of terrace walls for snails. *O. hupensis* were present in 38 of the 53 villages surveyed, found along irrigation ditches in 34 villages and terrace walls in 17 villages. Mean snail density by village was 2.3 snails/m^2^ in irrigation ditches (range: 0, 24.8 snails/m^2^) and 0.6 snails/m^2^ in terrace walls (range: 0, 8.5 snails/m^2^). The density of snails in terraces was not strongly correlated with snail densities in irrigation ditches (Spearman's rho 0.302).

Of the 7,325 snails collected from irrigation ditches, one infected snail was found. None of the 190 snails collected from terrace walls were infected.

### Evaluation of surveillance strategies

Acute case reporting and surveys for *S. japonicum*-infected snails yielded very low sensitivity: 1% and 3%, respectively (Table 5). Setting *n* to 500 and 5,000 yielded sensitivities of 3% and 0.3%, respectively for acute case reporting. The presence of snails in irrigation ditches yielded higher sensitivity (69%) and sensitivity was improved further when terraces were also sampled (74%); however specificity was low (44% and 33%, respectively). Surveys for *S. japonicum-*infected bovines had modest sensitivity (59%) and specificity (67%).

**Table 5:** The sensitivity and specificity of surveillance methods to identify villages with human *S. japonicum* infections.

	Human infections in village*		Sensitivity (95% CI)	Specificity (95% CI)	Percent of population tested
	Yes	No			
Hospital-based surveillance					
Acute schistosomiasis reported†					
Yes	9	2	1 (0.6–3)	99 (98–100)	NA
No	26	16			
Intermediate host and bovine monitoring					
*S. japonicum* infections in *O. hupensis*					
Yes	1	0	3 (0–10)	100	NA
No	34	18			
*O. hupensis* in irrigation ditches					
Yes	24	10	69 (53–83)	44 (21–69)	NA
No	11	8			
*O. hupensis* in irrigation ditches or terraces					
Yes	26	12	74 (59–88)	33 (11–56)	NA
No	9	6			
*S. japonicum* infections in bovines‡					
Yes	19	4	59 (43–77)	67 (36–100)	NA
No	13	8			
Targeted human testing					
*S. japonicum* infections in humans, aged ≥50 years					
Yes	27	0	77 (63–89)	100	39
No	8	18			
*S. japonicum* infections in households with bovines					
Yes	25	0	71 (55–85)	100	37
No	10	18			
Village-wide human testing					
*S. japonicum* infections in humans detected using the miracidium hatching tests**					
Yes	34	0	97 (90–100)	100	100
No	1	18			
*S. japonicum* infections in humans detected using Kato-Katz thick smear procedure††					
Yes	28	0	80 (66–93)	100	100
No	7	18			

*Village-wide infection surveys were used as the gold standard. Every resident was asked to provide three stool samples which were examined using the miracidium hatching test and Kato-Katz thick smear procedure.

**†:** Estimated sensitivity and specificity were adjusted to account for oversampling of villages where acute schistosomiasis was reported.

**‡:** Excludes six villages where bovines were present but not tested.

**Includes, for each person, three miracidium hatching tests from three stool samples.

**††:** Includes, for each person, three Kato-Katz slides prepared from one stool sample.

Testing only individuals who belong to high-risk groups provided the most accurate indicator of the presence of human infections in a village. Testing individuals aged 50 years and older or individuals in households that own bovines correctly identified 77% and 71% of villages where human infections were present, respectively. Specificity was 100%, as a single infected human was sufficient to designate a village as infected.

The use of only one of the two human infection testing methods led to decreased sensitivity. Testing all village residents using three hatching tests from three stool samples yielded a sensitivity of 97%. Testing all village residents by preparing three Kato-Katz slides from a single stool sample yielded a sensitivity of 80%.

## Discussion

Human *S. japonicum* infections were present in 35 of the 53 villages surveyed, with infection prevalence exceeding 20% in six villages, indicating widespread reemergence of schistosomiasis in areas where it had previously been controlled. Schistosomiasis was also detected in 13% of bovines, suggesting non-human mammalian reservoirs may play a role in the reemergence of schistosomiasis. Two key surveillance strategies, acute schistosomiasis reporting and surveys for *S. japonicum*-infected snails, grossly underestimated the number of villages where schistosomiasis had reemerged, misclassifying over 90% of villages where human infections were present. Alternative surveillance strategies, including surveys for the presence of the intermediate host snail or *S. japonicum* infections in bovine populations, yielded modest sensitivity and specificity. Testing high-risk human populations for *S. japonicum* infection appears to be the most reliable currently available strategy for monitoring the reemergence of human schistosomiasis, apart from testing all at-risk populations.

The reemergence of schistosomiasis documented here, in Sichuan province, and elsewhere underscores the challenge of sustaining reductions in human infections. Local elimination of human infections has proven difficult: a rise in *S. japonicum* infection prevalence was detected in endemic regions of China in the 2004 national infection survey [29]. The dramatic declines in *S. haematobium* and *S. mansoni* infection after widespread administration of praziquantel in West Africa were followed by an increase in infections in some areas [30]. China has developed a rigorous process for certifying reductions in infection, a process that accounts for spatial heterogeneity in transmission, the clustering of infections in few individuals and temporal variations in infection prevalence. To attain transmission control every endemic village in a county seeking certification must demonstrate that human infection prevalence is below 1% by testing at least 95% of the at-risk population. Villages are randomly selected for re-testing to confirm first-round results. All of the counties in this study had previously met transmission control criteria and therefore, every village in this study had, at one point, reduced infection prevalence below 1%. The fact that human infections were detected in 35 of these villages, at prevalences reaching 43%, highlights the instability of *S. japonicum* control, even when very low infection thresholds are attained, and the need for ongoing monitoring for reemergence.

The presence of at least 40 competent mammalian *S. japonicum* hosts, the potential for the parasite to be transported from endemic to controlled areas and the absence of a vaccine or lasting immunity contribute to the difficulty of local schistosomiasis elimination [10]. Bovine infections were present in 25 villages and bovine infection prevalence exceeded human infection prevalence in 18 of these villages, suggesting non-human reservoir species may be an important source of *S. japonicum* eggs in reemerging regions. The importance of different reservoir hosts appears to vary regionally. While previous studies have not found bovines to be important reservoirs in the endemic, hilly regions of China [31], [32] or in the Philippines [33], [34], in the hilly regions we studied, they may contribute to reemergence risk. Current efforts to replace bovines with tractors may reduce the risks posed by bovines [8], but, as rodents and dogs have been shown to play an important role in transmission in regions where bovines are absent [31], bovine removal may lead to a shift in the importance of other mammalian reservoirs. Connections between villages can sustain endemic schistosomiasis transmission and may promote the reintroduction of the parasite through social or hydrological networks [35]. In this study, schistosomiasis reemergence was detected the same year in two adjacent counties, and several villages with high human infection prevalence were located near each other on either side of the county line, suggesting infections may have spread across village and county boundaries. In addition, 34% of residents reported traveling outside of their villages in the past year, providing a possible mechanism for parasite import and export.

Treatment of human populations with praziquantel and the application of molluscicides were ongoing in the study region, likely prompted by the discovery of reemergence in these areas. This may explain why no human infections were detected in 7 of the 25 villages with historical evidence of reemergence and why snail populations were lower than observed elsewhere in Sichuan province [32]. However, the number of human and bovine infections detected in the region, despite ongoing treatment, underscores the need for improved surveillance.

Given the ongoing threat of schistosomiasis reemergence in controlled areas, timely and accurate detection of lapses in control are necessary to sustain disease control. Several types of surveillance methods have been used to monitor emerging and reemerging infections generally. First, infections may be detected through reporting of an acute presentation of a disease by health care providers. This has been a central strategy in monitoring poliomyelitis: acute flaccid paralysis, like acute schistosomiasis, is severe, rare – seen in approximately 1% of those infected with poliovirus – and occurs shortly after infection, providing a timely marker of renewed transmission [36]. China has a sophisticated disease reporting system [37], making surveillance for acute schistosomiasis appealing as it requires little additional capital or labor, although underreporting remains a concern. However, acute schistosomiasis reporting yielded poor sensitivity, identifying only 1% of villages where human *S. japonicum* infections were present.

Second, infections may be detected by monitoring non-human reservoirs, vectors or intermediate host populations. Non-human surveillance methods present the opportunity to detect the return of the parasite before human infections have occurred, a benefit that has been recognized in the design of surveillance systems to monitor emerging arboviruses [38], [39]. Snails, water buffalo, cows and other mammalian reservoirs have the potential to provide a key source of *S. japonicum* in reemerging regions and therefore may serve as sentinel populations. In our study regions, infected snails were rare, and therefore were a poor indicator of reemergence. Because we found only one infected snail out of over 7,000 examined in April 2007, the snail survey was repeated in half of the villages in September 2007. Again, only one infected snail was detected. Surveys for the presence of *O. hupensis* yielded modest sensitivity, but the method yielded numerous false positives. Given the low specificity, surveys for the presence of *O. hupensis* could be the first step in a two-part screening method that uses a more sensitive method to screen villages where snails are detected. The performance of surveys for the presence of *O. hupensis* may be different in regions where transmission has been controlled and snail control activities have been halted. Monitoring for the presence of *S. japonicum* infections in bovines offered modest sensitivity and specificity.

Third, surveillance may involve direct testing of human populations at high-risk of infection. Focusing human testing on a high-risk sample of the population as is currently being done to monitor the progress of lymphatic filariasis elimination [40], can increase the efficiency of surveillance in human populations, reducing the number of samples that need to be collected and tested. In this study, we identified two sentinel populations based on characteristics that can be readily identified by village leaders: older age and bovine ownership; that composed less than 40% of the total population. As this is the first population-level study of human *S. japonicum* infection in reemerging areas, research in other regions is needed to assess the generalizability of the high-risk characteristics we identified. However, our finding that infection prevalence was highest in older age groups is consistent with other studies of *S. japonicum* in China and the Philippines [29], [41], and supports our conclusion that children make poor surveillance targets for *S. japonicum*. In contrast, *S. haematobium* and *S. mansoni* infections typically concentrate in children and teenagers [42] suggesting that sentinel age groups may vary across schistosome species. Bovines have been shown to play a key role in endemic schistosomiasis transmission in the lakes and marshland regions of China [43], [44], suggesting bovine ownership may also indicate human reemergence risk in other regions, but this may not be the case in the Philippines [33]. Schistosomiasis is strongly associated with poverty, thus it is not surprising that higher educational attainment appeared protective against *S. japonicum* infection [45], [46]. However, as with rapeseed planting, the highest risk groups comprised over 50% of the population, leading to minimal gains in efficiency.

High-risk human population monitoring was the most accurate alternative to testing all at-risk people, outperforming bovine and snail-based surveillance as well as acute schistosomiasis reporting. Longitudinal studies, currently underway, will aid in identifying appropriate sampling intervals, as will mathematical models of reemergence over time and space. An analysis of the costs-effectiveness of different surveillance strategies can further aid in the development of an effective and feasible surveillance protocol in areas approaching schistosomiasis elimination.

Sentinel human populations may also be defined by geographic and environmental characteristics. Even within a relatively homogeneous region in terms of demographic characteristics and disease control measures, infection prevalence and intensity may vary widely between villages, as observed in this study and elsewhere [32], [47]. Local variations in factors such as rainfall, sanitation and intermediate host habitat may promote or impede the acquisition of human parasitic infections and ultimately, the reemergence of human infections [48], [49]. The characterization of local environments that are at high risk of schistosomiasis reemergence can further refine the definition of human surveillance targets.

As schistosomiasis infections decline, highly sensitive diagnostic tests will be needed to identify remaining infections. We used two stool-based testing methods, the Kato-Katz thick smear procedure and the miracidium hatching test to identify human infections. These methods are highly specific but, due to variability in egg excretion by infected individuals, the sensitivities of these tests decline when infection intensity is low [50], [51]. The use of multiple diagnostic tests and the collection of stool samples on multiple days increase the likelihood of detecting infections. Nonetheless, the infection prevalences detected here are likely lower bound estimates. PCR-based methods to detect schistosome eggs in the stool of human and other mammalian hosts show promise and may provide the sensitivity needed to diagnose very low intensity *S. japonicum* infections in regions approaching schistosomiasis elimination [52], [53]. Similarly, PCR-based methods to identify the presence of cercariae in water may aid in the identification of environments where the parasite remains endemic [54]. Pooled analysis of snails, water samples or even mammalian stool for *S. japonicum* may increase the efficiency of population-based surveillance at low infection intensities [55].

The sensitivity and specificity estimates presented here are calculated for the methods as we performed them. For example, surveys for the presence of *O. hupensis* in irrigation ditches were conducted by sampling all irrigation ditches in a village at 10 m intervals – if fewer locations are sampled, the probability of finding the snail host, and therefore the sensitivity of the test, may be diminished. Similarly, immunoassays are often used to screen humans for *S. japonicum* infection in China, and, due to the low specificity of this assay, individuals with a positive immunoassay result are then asked to provide a stool sample for examination using the miracidium hatching or Kato-Katz test, as was done in the 2004 national schistosomiasis survey [29]. As individuals with a negative immunoassay are not screened using coprological methods, this two-step screening produces false negatives from both diagnostic methods, yielding an overall sensitivity lower than that of the immunoassay alone. Targeted sampling of high-risk populations, using an immunoassay based method will yield different sensitivities than those calculated here. Similarly, relative distributions of *S. japonicum* infections in bovine, snail and human populations may vary regionally, a factor that should be considered when adopting post-control surveillance plans.

We attempted to test all humans and bovines in the 53 selected villages for infection, but approximately 30% of the population did not participate. Estimating the true population of each study village, and therefore the true infection survey participation percentage, is difficult. Due to residency requirements, government population registers in rural areas often include families that have moved to urban areas without registering such moves. Conversations with village leaders suggest almost all residents who spent most of their time in the village were captured by the demographic and household surveys. Participation in the infection surveys was lowest among people who spent time out of their village in the past year and young adults. Among those tested, neither young adults nor people who left their village had high infection prevalence. Nonetheless, it is possible that some infections were missed, leading some villages where infections were present only among non-participating residents to be misclassified. The number of villages with human or bovine infections may be greater than reported here.

The dramatic reduction of schistosomiasis in China and elsewhere has prompted consideration of the next phase of schistosomiasis control, motivating public health leaders to look beyond morbidity control toward the elimination of human schistosomiasis [1], [8], [21]. While this transition marks progress in controlling schistosomiasis, new challenges arise when approaching elimination. The reemergence of schistosomiasis as documented here highlights the transience of reductions in schistosomiasis in some areas. Before the introduction of praziquantel, schistosomiasis control focused on environmental modifications to reduce snail habitat and improve sanitation [2], [56]. The long-term interruption of schistosomiasis transmission in China and elsewhere will require the integration of praziquantel treatment and alterations to local environments to reduce their potential to sustain the parasite lifecycle [49], [57]. In addition, it will require a surveillance system that can detect the reemergence of infections with sufficient speed and accuracy to allow interventions to halt renewed transmission and prevent the further spread of infections.

## Supporting Information

Checklist S1 STROBE Checklist.
(0.02 MB PDF)
